# An Unusual Presentation of Pediatric Conjunctival Mucosa-Associated Lymphoid Tissue Lymphoma

**DOI:** 10.1155/2019/4061368

**Published:** 2019-04-30

**Authors:** Samantha Bobba, Christopher Go, Amanda Charlton, James Smith, Maciek Kuzniarz, Subhashini Kadappu

**Affiliations:** ^1^University of Sydney, Sydney, Australia; ^2^Sydney Eye Hospital, Sydney, Australia; ^3^LabPLUS, Auckland City Hospital, Auckland, New Zealand; ^4^Westmead Children's Hospital, Sydney, Australia; ^5^EyeVision Canberra, Australia

## Abstract

Ocular adnexal mucosa-associated lymphoid tissue (MALT) lymphoma is uncommon in the pediatric population. Initial misdiagnosis is common and there is lacking consensus regarding the optimal approach to treatment. Herein, we report an atypical presentation of pediatric conjunctival MALT lymphoma and review the presentation and management of this rare condition.

## 1. Introduction

Whilst ocular involvement occurs in only one to two percent of extranodal non-Hodgkin Lymphomas (NHLs), mucosa-associated lymphoid tissue (MALT) lymphoma is the most common type of primary ocular adnexal lymphoma [1]. First described by Issac and Wright (1983), MALT lymphoma is characterized by the presence of small B-cell lymphocytes of low-grade malignancy [2]. MALT lymphoma most commonly involves the gastrointestinal tract, salivary gland, lung, and thyroid gland. It typically affects patients in their fifth to seventh decades, with females at higher risk than males. MALT lymphoma usually has an indolent course, which is highly responsive to radiotherapy [3].

There are no accepted prognostic factors for MALT lymphoma of the ocular adnexal region. Several chromosomal abnormalities have been demonstrated in MALT lymphoma more generally. A potential association between ocular MALT lymphoma and* Chlamydia psittaci *has been suggested but no definitive infectious etiology or association has been identified [4]. This is in contrast to gastric MALT lymphoma, in which* Helicobacter pylori *has been shown to be the causative agent in the majority of cases [5].

Conjunctival MALT lymphoma characteristically manifests as a painless fleshy-coloured “salmon-patch” lesion arising from the fornix [6]. Bulbar involvement is more common, usually more easily recognized, and is associated with a better prognosis than MALT lymphoma of the palpebral conjunctiva. Biopsy is critical to diagnosis, utilizing specific morphologic histological features and immunohistochemical markers [3]. Clinically, it can be difficult to differentiate between reactive lymphoid hyperplasia and MALT lymphoma of the ocular adnexa, and molecular analysis and histopathology do not always correlate [6]. In ambiguous cases, assessing responsiveness to a short course of antiallergenic treatment may aid in the diagnosis of patients presenting with a conjunctival “salmon-patch” lesion.

Conjunctival MALT lymphoma is rare in children, with the first case reported by Tiemann and colleagues (2004) just over a decade ago [7]. To date, only a handful of pediatric cases have been published, summarized in Table 1 [1, 6–13]. Herein, we report an atypical case of conjunctival MALT lymphoma in a 15-year-old patient, presenting without the “salmon-patch” lesion that typically characterizes the condition.

## 2. Case Report

**Figure 1:**
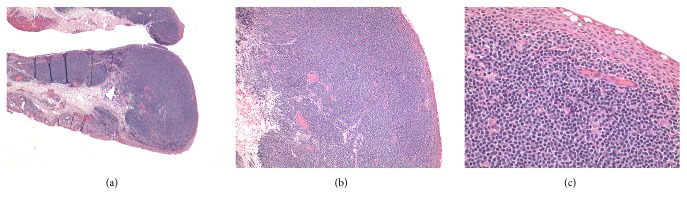
*Initial biopsy of the patient's right palpebral conjunctival lesion*. (a) Low magnification overview showing expansion of the subepithelial connective tissue by coalescent nodules of lymphocytes forming a mass lesion. H&E stain. (b) Medium magnification showing a mass forming sheet of small mature monomorphic lymphocytes compatible with a neoplastic process and not the mixed lymphocyte morphology of reactive lymphoid follicles. H&E stain. (c) High magnification showing monomorphic centrocyte-like and small mature lymphocytes without mitotic figures or tingible body macrophages, indicating a low-grade lymphoma. H&E stain.

A 15-year-old female presented with a one-year history of intermittent bilateral ocular erythema, irritation, and discomfort, most severe in the right eye. She was otherwise well, with no significant past medical history or family history. Visual acuity was 6/6 in both eyes. On slit-lamp examination, giant papillae were identified bilaterally in the inferior conjunctival forniceal regions, notably larger and more widespread in the right eye (Figure 1). Baseline blood tests including liver function, electrolytes, and full blood count were in normal range. The patient was initially diagnosed with allergic conjunctivitis. Whilst her ocular erythema improved with topical steroids, she experienced persistent irritation and discomfort of the right eye and represented three months later.

A biopsy of the right palpebral conjunctival lesion showed expansion of the subepithelial connective tissue by coalescent nodules of small lymphocytes. These lymphocytes had a centrocyte-like morphology; the immunophenotype is CD20+/CD10-/CD5-/CD43-. The cell markers on flow cytometry showed a monoclonal population of mature B cells with lambda light chain restriction. The morphology and immunophenotype, including immunoglobulin light chain restriction, were diagnostic of an extranodal marginal zone lymphoma of the mucosa-associated lymphoid tissue (MALT lymphoma) (Figure 1). Notably, the patient's ocular examination had been atypical of the “salmon-patch” appearance that is characteristic of the condition. Lumbar puncture, bone marrow trephine, whole-body positron emission tomography scanning, and magnetic resonance imaging of the brain did not reveal any abnormalities to suggest lymphoma outside the ocular adnexal tissue. The patient was managed with a total of ten interferon alpha-2 beta injections (ten million units per dose) into the conjunctival fornix over a three-month period, evenly distributed over this time period (i.e., administered at approximately weekly intervals). Posttreatment biopsy five weeks later demonstrated reactive lymphoid hyperplasia with no clonal B cells on flow cytometry. Clinical resolution of symptoms was observed within two months of completing treatment, with no signs of recurrence up to eight years after treatment.

## 3. Discussion

Primary ocular adnexal lymphoma is rare in children, and thus the majority of data regarding the condition is obtained from adult populations, [6]. Whilst various case series of ocular adnexal lymphomas include pediatric patients, they rarely specify details such as the patients' presenting symptoms, diagnosis, or management approaches, which are critical to determining the course of pediatric conjunctival MALT lymphoma. Systemic review of Ovid MEDLINE, EMBASE, and PubMed databases (last searched 1^st^ December 2017, key words “conjunctival” OR “ocular” and “MALT lymphoma” and “paediatric/pediatric” or “child”) identified 10-13 pediatric cases of conjunctival MALT lymphoma overall (summarized in Table 1) [1, 6–13], with specific details on the presentation, management, and follow-up reported in only five cases [1, 7–10].

Conjunctival MALT lymphoma typically presents with the characteristic “salmon-patch” lesion [4], albeit with varying clinical symptoms reported in the literature [6]. To the best of our knowledge, this is the first pediatric case of conjunctival MALT lymphoma diagnosed in the absence of a “salmon-patch” lesion. Notably, Lucas and colleagues (2003) first reported a 15-year-old male presenting with an eight-month history of small follicular deposits in the conjunctival nasal fornices, without a “salmon-patch” lesion. Whilst flow cytometry was somewhat convincing of a low-grade B-cell lymphoma, absolute distinction of lymphoma type was not possible due to the small amount of tissue obtained at biopsy [14]. The lack of a “salmon-patch” lesion and involvement of the palpebral rather than bulbar conjunctiva in our case report highlights the importance of exercising caution in pediatric patients with persistent conjunctivitis, even if the initial presentation appears typical of an allergic or viral etiology.

Tiemann and colleagues (2004) are largely credited with reporting the first definitive case of conjunctival MALT lymphoma in a ten-year-old girl who was successfully managed with surgical excision of the lesion and adjuvant local cryotherapy [7]. Since then, alternate treatment modalities have included topical interferon, local radiotherapy and chemotherapy, consistent with common management approaches in the adult population (Table 1) [1, 6–12, 15, 16]. Of the ocular adnexal lymphomas, conjunctival lesions lend themselves to localized therapy, as they are the least likely to involve disseminated disease [9]. Some, however, suggest that combined radiotherapy and chemotherapy is preferable due to the potential for local relapse [10, 14]. Local radiotherapy is often favored in adults given the high responsiveness of MALT lymphoma to radiotherapy [3, 16]. However, potential complications of radiotherapy and chemotherapy, such as deformities of the orbit, cataracts, secondary malignancy, and corneal ulceration, may outweigh the treatment benefits in children [9]. Systemic immunotherapy with anti-CD20 monoclonal antibodies, namely, rituximab, has also demonstrated success in achieving complete remission in patients with MALT ocular adnexal lymphomas [17]. Other potential novel biological agents in treating MALT lymphoma include ibrutinib, which has demonstrated success in selected case reports of refractory MALT lymphoma [18, 19]. Topical interferon therapy has recently emerged as an alternate option that modulates immune responses and affects cell proliferation [8, 9, 14, 15]. In the literature, it is typically administered once to twice weekly over a one-three-month period. Nonsight threatening complications such as chemosis and subconjunctival haemorrhage have been reported, in addition to transient systemic adverse effects including flu-like illness with fevers, chills, myalgias, headaches, and nausea [14]. Overall, the adverse effects reported to date have been relatively minor. Our case demonstrates the success of intralesional interferon-*α*-2b as a monotherapy in inducing long-term remission. Importantly, we also report the longest duration of follow-up to date, almost triple that in previous studies. Given that both local relapse and delayed systemic manifestations of ocular MALT lymphoma have been reported, long-term surveillance of the condition is critical, particularly in pediatric patients.

## 4. Take-Home Messages

- An atypical case of pediatric MALT lymphoma involving the palpebral conjunctiva is presented, different from the characteristic “salmon-patch”, which typically affects the bulbar conjunctiva.
- Initial misdiagnosis suggests caution should be taken in pediatric patients presenting with atypical persistent conjunctivitis.
- Although the risk of systemic involvement is low, long-term follow-up in children is important and was significantly greater in this case than previously reported.
- This case report and review also demonstrate the long-term benefits of topical interferon treatment as a monotherapy.

## Figures and Tables

**Table 1:** Summary of comprehensive case reports of paediatric conjunctival MALT lymphoma.

Case/Series	Age, Sex	Presentation	Management	Duration of follow-up *(in remission unless otherwise specified)*
Tiemann et al., 2004	10, F	painless swelling of eye, “salmon-patch” lesion	Surgical excision and local cryotherapy	15 months

Chan et al., 2004	10, ∗	∗	∗ (no radiation or chemotherapy)	Bilateral recurrence at 6 months

Claviez et al., 2006	11, F	∗	Surgical excision and local cryotherapy	2.5 years

Fuentes-Paez et al., 2007	5, F	purulent ocular secretions, photophobia, “salmon-patch” lesion	Topical interferon a6	1 year

Ferry et al., 2007	∗3 cases aged < 21yro in study of 353 patients. However, no information regarding whether the conjunctiva or ocular adnexa were affected.			

Holds et al., 2010	14, F	eyelid swelling, ptosis, tearing, enlarging “salmon-patch” lesion	Intralesional interferon-*α*-2b	27 months

Frenkel et al., 2010	13, M	irritation, blurred vision, inability to close eyes due to enlarging “salmon-patch” lesion	Intravenous Rituximab	14 months

Incesoy-Ozdemir et al., 2014	10, M	asymptomatic conjunctival mass	Local radiotherapy	4 years

Beykin et al., 2014	16, ∗	“salmon-patch” lesion, nil other details	∗	82 months

Beykin et al., 2014	17, ∗	“salmon-patch” lesion, nil other details	∗	82 months

Hoh et al., 2016	13, M	Salmon-coloured, bulging, elastic tumour filling entire lower conjunctival fornix	Systemic doxycycline and azithromycin for 10 months	5 years

*∗*Missing data.
